# Increasing incidence of hemorrhagic fever with renal syndrome could be associated with livestock husbandry in Changchun, Northeastern China

**DOI:** 10.1186/1471-2334-14-301

**Published:** 2014-06-03

**Authors:** Jing Wu, Dan-Dan Wang, Xin-Lou Li, Sake J de Vlas, Ya-Qin Yu, Jian Zhu, Ying Zhang, Bo Wang, Li Yan, Li-Qun Fang, Ya-Wen Liu, Wu-Chun Cao

**Affiliations:** 1 Department of Epidemiology and Statistics, Jilin University, Changchun, People’s Republic of China; 2 Changchun Center for Disease Control and Prevention, Changchun, People’s Republic of China; 3 School of Public Health, Central South University, Changsha, People’s Republic of China; 4 State Key Laboratory of Pathogens and Biosecurity, Beijing Institute of Microbiology and Epidemiology, Beijing, People’s Republic of China; 5 Department of Public Health, Erasmus MC, Rotterdam, The Netherlands

## Abstract

**Background:**

Since the end of the 1990s, the incidence of hemorrhagic fever with renal syndrome (HFRS) has been increasing dramatically in Changchun, northeastern China. However, it is unknown which, and how, underlying risk factors have been involved in the reemergence of the disease.

**Methods:**

Data on HFRS cases at the county scale were collected from 1998 to 2012. Data on livestock husbandry including the numbers of large animals (cattle, horses, donkeys and mules), sheep, and deer, and on climatic and land cover variables were also collected. Epidemiological features, including the spatial, temporal and human patterns of disease were characterized. The potential factors related to spatial heterogeneity and temporal trends were analyzed using standard and time-series Poisson regression analysis, respectively.

**Results:**

Annual incidence varied among the 10 counties. Shuangyang County in southeastern Changchun had the highest number of cases (1,525 cases; 35.9% of all cases), but its population only accounted for 5.6% of the total population. Based on seasonal pattern in HFRS incidence, two epidemic phases were identified. One was a single epidemic peak at the end of each year from 1988 to 1997 and the other consisted of dual epidemic peaks at both the end and the beginning of each year from 1998 to the end of the study period. HFRS incidence was higher in males compared to females, and most of the HFRS cases occurred in peasant populations. The results of the Poisson regression analysis indicated that the spatial distribution and the increasing incidence of HFRS were significantly associated with livestock husbandry and climate factors, particularly with deer cultivation.

**Conclusions:**

Our results indicate that the re-emergence of HFRS in Changchun has been accompanied by changing seasonal patterns over the past 25 years. Integrated measures focusing on areas related to local livestock husbandry could be helpful for the prevention and control of HFRS.

## Background

Hemorrhagic fever with renal syndrome (HFRS) is one of the rodent-borne diseases caused by a hantavirus (family *Bunyaviridae*). HFRS mainly includes epidemic hemorrhagic fever (EHF), which mostly occurs in Asia, and nephropathis epidemica (NE), which mostly occurs in Europe [1,2]. The typical clinical symptoms of HFRS are fever, hemorrhage, headache, back pain, abdominal pain, acute renal dysfunction, and hypotension. Human infections result from inhalation of aerosols contaminated by hantavirus shed in excreta, saliva, and urine of infected rodents [3,4]. Approximately 70% to 90% of the HFRS cases worldwide have been reported in mainland China [5]. Most of these cases were caused by one of two hantaviruses, the Hantaan virus (HTNV) and the Seoul virus (SEOV). HTNV and SEOV are mainly carried by *Apodemus agrarius* (striped field mouse) and *Rattus norvegicus* (Norway rat), respectively [6-8]. For patients infected with HTNV, the clinical manifestations are more severe and the fatality rate is usually higher compared to SEOV infection [1,9]. *A. agrarius* are mostly found in farm field habitats, and *R. norvegicus* lives in or around human environments (e.g., residences, warehouses and stores). HFRS cases caused by HTNV usually peak during the fall-winter period, and most of the SEOV-associated HFRS cases are reported during the spring months [10-12]. During the past decade, the overall HFRS incidence has declined considerably in mainland China, from 3.05 per 100,000 population to 0.84 per 100,000 population (>3-fold). However, the proportion of HFRS resulting from SEOV infections continues to expand [10,13]. During the four decades after the first HFRS case was reported in 1955 in Jilin Province, there was a low level of HFRS endemicity in Jilin. However, the HFRS incidence has increased significantly since the end of the 1990s, especially in Changchun, the capital of the province [14]. The temporal and spatial patterns and potential factors underlying the reemergence of the disease remain unclear. The objectives of our study were to explore the spatial and seasonal patterns of HFRS distribution for different epidemic phases, and to study the association between HFRS incidence and livestock husbandry, climate factors, and land cover.

## Methods

### Study area

**Figure 1:**
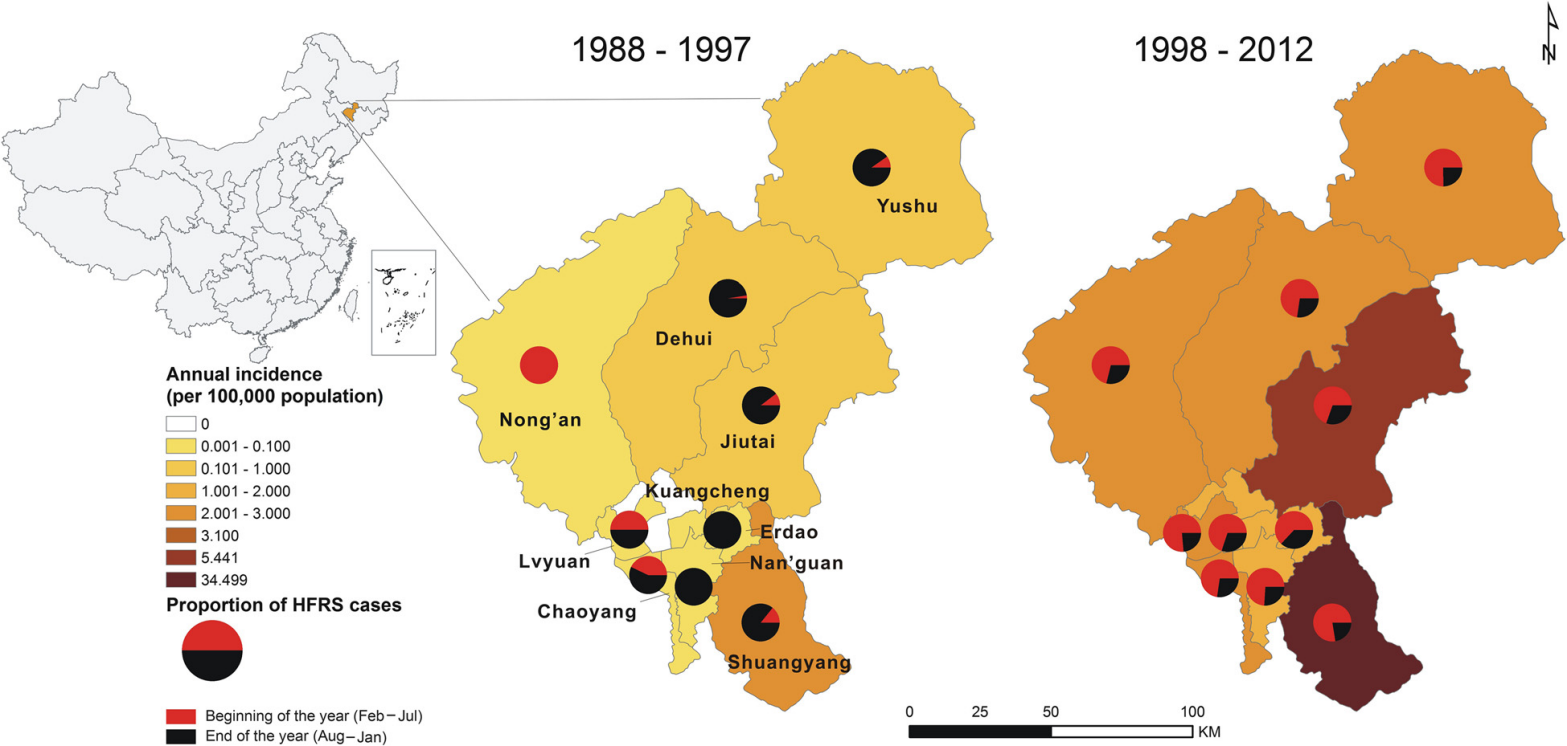
**Thematic map of annual incidence for each county before and since 1998, Changchun.** The gradient colors represent HFRS incidence for each county, and the pie-charts with red and black colors indicate the proportions of HFRS cases for two 6-month study periods (beginning of the year (February–July) and end of the year (August–January)).

The research area covered Changchun (43.26° to 45.3° north latitude, 124.5° to 127.2° east longitude), an inland prefecture in Jilin Province, northeastern China (Figure 1). Changchun has 7.6 million residents in 10 counties with a total land area of 20,660 km^2^. The counties of Chaoyang, Lvyuan, Nan’guan, Kuancheng and Erdao are considered to be urban areas. The other five counties (i.e., Shuangyang, Jiutai, Nong’an, Yushu and Dehui) are rural areas. Yushu, Dehui, Jiutai and Nong’an counties are important depots for rice and corn. Livestock husbandry has an important economic role in Changchun, especially sika deer (*Cervus nippon*) cultivation. The numbers of farms that cultivate sika deer and other animals have increased in Changchun since the 1990s.

### Data collection and management

HFRS has been a Class B Notifiable Infectious Disease since the 1950s. A standard protocol developed by the Chinese Center for Disease Control and Prevention has been used for reporting of the disease [15]. Prior to 1983, HFRS cases were clinically diagnosed according to the National Guideline [16]. The clinical diagnostic criteria included: exposure history (i.e., field work experience and exposure to rodents and their excreta, saliva, and urine within 1–6 weeks prior to the onset of illness); acute illness with at least two of eight (i.e., fever, chill, hemorrhage, headache, back pain, abdominal pain, acute renal dysfunction, hypotension) clinical symptoms; congestion, edema, bleeding tendency and abnormity of blood and urine routine parameters; and experience or partial experience with the five phases (i.e. fever, hypopiesis, oliguresis, hyperdiuresis, and recovery) of the disease [15]. Since 1983, HFRS cases have been diagnosed clinically using signs and symptoms and also confirmed using antibody-based serological tests (e.g. MacELISA, IFA), hantavirus isolation, or detection of hantavirus RNA (e.g. RT-PCR) in serum samples from the patient [13,15,16].

The first HFRS case was reported in 1959 in Changchun, which is now a national HFRS surveillance site [15]. Data that included all 4,251 of the HFRS cases that were reported in Changchun during 1988–2012 were collected from the ten county centers for disease control and prevention (CDCs). To calculate the annual incidence, annual demographic data for the 1988 to 2012 period were collected from the Changchun Bureau of Statistics. All personal data were anonymous, and were publicly available secondary data.

The study protocols were reviewed and approved by the research institutional review board (IRB) of Changchun CDC and the Beijing Institute of Microbiology and Epidemiology. Readers interested in further research can contact the corresponding author to obtain the full dataset used in this study.

### Analysis of epidemiologic patterns of human HFRS cases

The thematic maps of annual incidence before and since 1998 were created in gradient colors to display the spatial distribution of HFRS incidence in each county of Changchun (ArcGIS 9.3 software, ESRI Inc., Redlands, CA, USA). To illustrate the seasonal distribution of HFRS cases for each county, the proportions of HFRS cases for two 6-month study periods (beginning of the year (February–July) and end of the year (August–January)) were indicated by red and black pie charts overlapping the corresponding counties on the map. The bar charts of monthly HFRS incidence in Shuangyang County (the HFRS epidemic hotspot in Changchun), and in the other nine counties combined, over a 25-year period were created to display the seasonal incidence pattern of HFRS. To consider the different seasonal incidence patterns of HFRS caused by HTNV and SEOV, the red and black colors were also used to present the monthly incidence for the periods February–July and August–January, respectively. Bar charts of average incidence by age and gender, and pie charts of case proportions by occupation, before and since 1998, were also created.

### Statistical analysis

To explore risk factors that affected the spatial heterogeneity of HFRS incidence in Changchun, annual data on livestock husbandry that included the numbers of large animals (cattle, horses, donkeys and mules), sheep, and deer during 1988–2012 were collected for each county from the Annual Statistic Report of Changchun. The annual densities of these animal populations were calculated from the data. Annual climatic data that included annual mean temperature, total precipitation and mean relative humidity for the 1988 to 2012 period were collected for each county from the China Meteorological Data Sharing Service System ( http://cdc.cma.gov.cn). To extract the percent coverage by croplands, forests, grassland, and built-up lands for each county, data on Changchun land cover (1990, 1995, 2000, 2005, and 2010) were collected from Chinese data-sharing network of earth-system science ( http://www.geodata.cn/) and the Northeast Institute of Geography and Agroecology, Chinese Academy of Sciences [17,18]. To explore the different epidemic patterns of HFRS incidence before and since 1998, a separate Poisson regression analysis was performed for each of the two phases. The cumulative number of HFRS cases for each county was set as the outcome variable, and population number was included as the offset variable. For each county, potential risk factors for the two phases (e.g., livestock husbandry (average density of large animals, sheep, and deer), climate factors (average temperature, precipitation, and relative humidity) and land cover (average percent coverage by croplands, forests, grassland, and built-up lands)) were included as co-variables in the analysis. The percentage change (PC) in incidence in response to a change in a variable by a given amount (100*(exp(coefficient)-1)), 95% confidence intervals (CIs), and P-values were estimated after correction for over-dispersion. For PC estimation, a 10 head/km^2^ difference was used for monthly livestock husbandry, and 1°C, 1 mm, and 1% differences were used for annual mean temperature, total precipitation and mean relative humidity, respectively. A 1% difference was used for land cover. Univariate analyses were performed to examine the effects of individual variables. Multivariate analyses were then performed using the variables from the univariate analysis with a P-value < 0.1 as covariates. Correlations between covariates were quantitatively assessed and models were optimized by comparing -2 log likelihood results when correlated variables were added or removed one by one. STATA statistical software (Version 10.0, StataCorp LP, Texas, USA) was used in the analysis.

To explore the factors affecting the reemergence of HFRS in Changchun, time series Poisson regression analyses were performed for the 1988–2012 period using the Shuangyang County data, and the combined data from the other nine counties, respectively. Time-series Poisson regression deals with the annual number of HFRS cases. The inclusion of annual population size as an offset variable allows for an analysis of incidence. Similar to the approach described above, multivariate analyses were performed by including all variables from the univariate analyses with P-values < 0.10. Correlations between covariates were quantitatively assessed and models were optimized by comparing-2 log likelihood results when correlated variables were added or removed one by one. The PCs that represented the associations between HFRS cases and determinants were derived from the final model. Each PC estimate included a 95% CI and a P-value. We also adjusted for the effects of improved measures of prevention and control on HFRS incidence since 2005. STATA statistical software was also used in this analysis.

## Results

### The distribution patterns of HFRS incidence in space, season, and human populations

**Figure 2:**
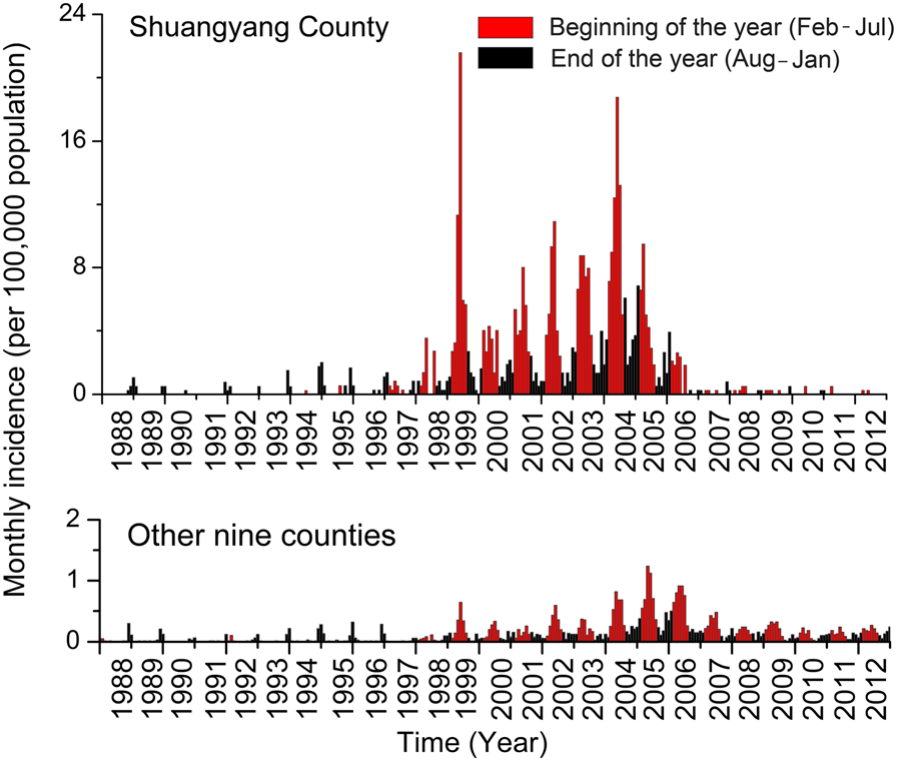
**Temporal distribution of monthly HFRS incidence in Shuangyang County and of the combined monthly incidence totals for the other nine counties in Changchun.** The upper and lower panels represent the monthly incidence in Shuangyang County and the combined total monthly incidence for other nine counties, respectively. Red and black colors indicate monthly incidence for the February–July and August–January periods, respectively.

**Figure 3:**
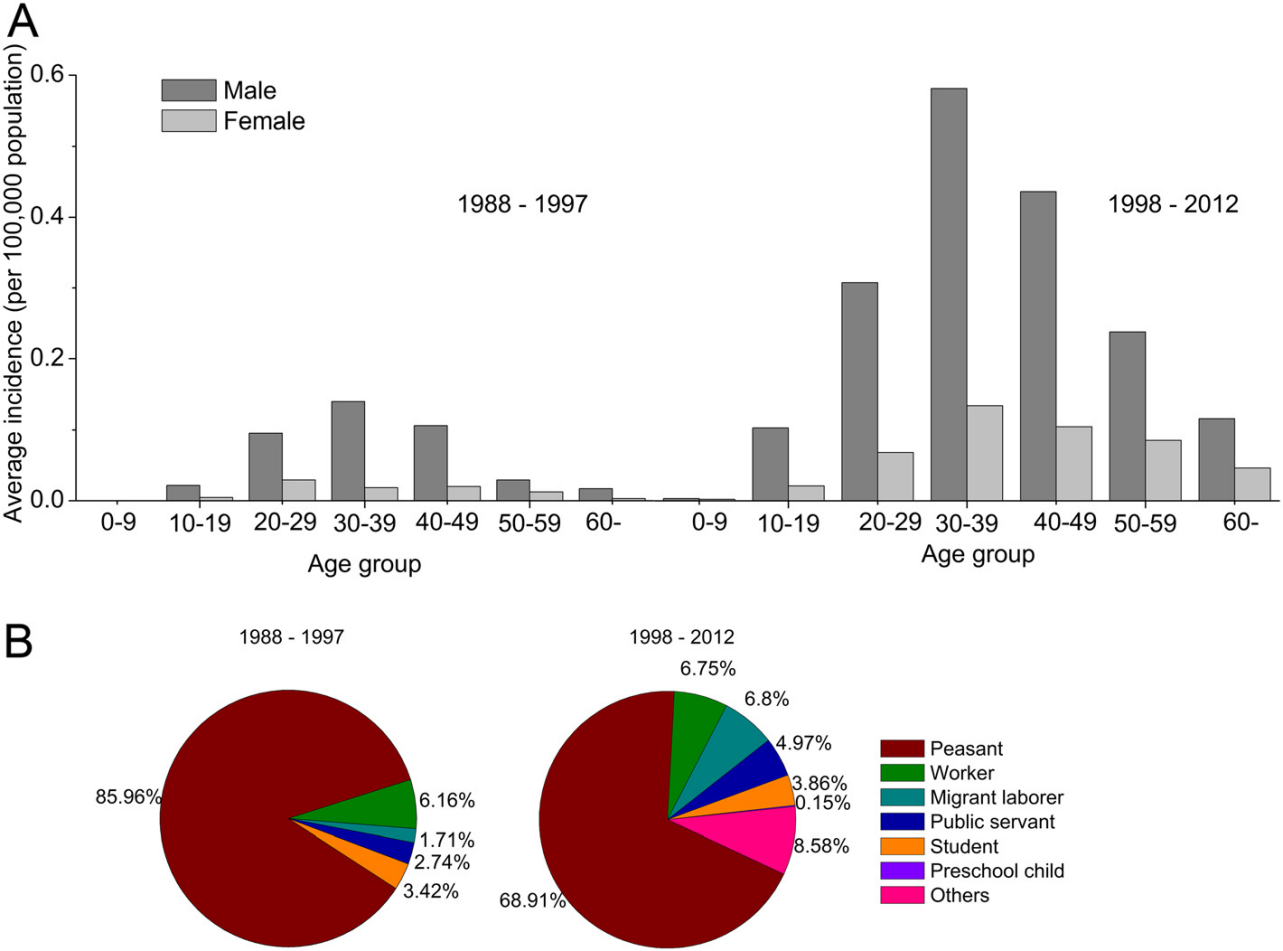
**Age, gender, and occupational distribution of reported HFRS cases. (A)** Average incidence over age groups and sex during two epidemiological phases (1988–1997 and 1998–2012). **(B)** Occupational proportions for HFRS cases during the two phases. Peasants indicate people engaged in farming or livestock breeding; workers indicate people who work in manufacturing; students are grade school pupils, high school students, and undergraduates; public servants are teachers, doctors, civil servants, and individuals retired from these occupations; migrant laborers are migrant workers, restaurant servers, shop workers and housekeepers; preschool child indicates children < 7 years of age.

A total of 4,251 HFRS cases were reported during 1988–2012. Cases were distributed throughout the 10 counties of Changchun. The annual incidences varied greatly among the ten counties. Shuangyang County in southeastern Changchun had the highest annual incidence, both before and since 1998 (Figure 1). The Shuangyang County cases accounted for 35.9% (1,525/4,251) of all cases in the entire city during the 25-year period, but the population of the county only accounted for 5.6% of the total Changchun population. There were also seasonal changes in the proportion of HFRS cases in most counties (Figure 1). Based on seasonal shifts in HFRS incidence in 1997, the temporal pattern of HFRS incidence could be characterized by two phases. The Phase I pattern occurred in 1988 to 1997, with a typical peak in incidence at the end of each year (black bar, Figure 2). This pattern represented a seasonal characteristic of HTNV transmission. The Phase II pattern occurred in 1998 to 2012, with an emerging peak in incidence at the beginning of each year (red bar, Figure 2) that reflected a pattern characteristic of SEOV transmission. As incidence rapidly increased since 1998, the peak in incidence at the beginning of each year became dominant. In Shuangyang County, dual peaks of incidence were clearly evident during phase II. A rapid increase (of approximately 10 times) in HFRS incidence occurred at the beginning of each year and a smaller increase in incidence occurred at the end of each year (Figure 2). After 10 years of age, incidence was higher in males compared with females for each age group. In phase I and phase II, 85.96% and 68.91%, respectively, of the patients were from the peasant population. The proportion of patients from the migrant laborer population increased from 1.71% to 6.80% during the two phases (Figure 3).

### Factors relating to spatial heterogeneity in HFRS incidence

The results of the univariate analyses indicated that during phase I, the spatial distribution of HFRS incidence was associated with annual precipitation and average relative humidity (P < 0.05), and HFRS incidence was related although not significantly also to deer density (P = 0.057). The results of multivariate analysis indicated that HFRS incidence was associated only with average relative humidity, the two variables increasing together. Univariate analysis revealed that during phase II, the spatial distribution of HFRS incidence was associated with deer density and annual precipitation (P < 0.05), but HFRS incidence was associated only with deer density in the multivariate analysis. The incidence increased as the deer density increased. HFRS incidence was not significantly associated with land cover in either phase (Table 1).

**Table 1:** Factors affecting spatial heterogeneity in HFRS incidence in Changchun

**Period**	**Variable (unit)**	**Univariate analysis**		**Multivariate analysis**	
		**PC (95% CI)**	**P-value**	**PC (95% CI)**	**P-value**
**1988-1997**	**Livestock husbandry**				
	Large animals (10 h/km^2^)	60.5 (-68.9, 729.4)	0.572	-	-
	Sheep density (10 h/km^2^)	-41.3 (-81.6, 87.4)	0.368	-	-
	Deer density (10 h/km^2^)	154.6 (-2.6, 565.4)	0.057	NS.	NS.
	**Climate factors**				
	Temperature (1°C)	-74.6 (-95.6, 48.0)	0.128	-	-
	Precipitation (1 mm)	26.7 (2.3, 56.9)	0.030	NS.	NS.
	Relative humidity (%)	243.1 (136.3, 398.1)	< 0.001	243.1 (136.3, 398.1)	< 0.001
	**Land cover**				
	Irrigated cropland (1%)	16.7 (-2.0, 38.9)	0.084	NS.	NS.
	Rainfed cropland (1%)	-2.8 (-11.8, 7.0)	0.564	-	-
	Forest (1%)	4.5 (-4.4, 14.3)	0.332	-	-
	Grassland (1%)	-28.8 (-63.9, 40.4)	0.327	-	-
	Built-up lands (1%)	-25.2 (-54.0, 21.6)	0.242	-	-
**1998-2012**	**Livestock husbandry**				
	Large animals (10 h/km^2)^	2.1 (-21.2, 32.3)	0.874	-	-
	Sheep density (10 h/km^2^)	-12.1 (-43.8, 37.4)	0.571	-	-
	Deer density (10 h/km^2^)	41.4 (30.2, 53.5)	< 0.001	41.4 (30.2, 53.5)	< 0.001
	**Climate factors**				
	Temperature (1°C)	7.7 (-87.6, 836.5)	0.947	-	-
	Precipitation (1 mm)	28.6 (7.8, 53.4)	0.005	NS.	NS.
	Relative humidity (%)	46.3 (-19.5, 165.7)	0.211	-	-
	**Land cover**				
	Irrigated cropland (1%)	6.4 (-7.7, 22.6)	0.392	-	-
	Rainfed cropland (1%)	-2.4 (-10.6, 6.5)	0.580	-	-
	Forest (1%)	4.4 (-4.4, 14.0)	0.342	-	-
	Grassland (1%)	-17.1 (-52.6, 45.0)	0.511	-	-
	Built-up lands (1%)	-3.5 (-15.2, 9.8)	0.591	-	-

Poisson regression analysis results after correction for over-dispersion.

### Factors affecting temporal trends in HFRS incidence

For Shuangyang County, the results of the univariate time-series Poisson analysis revealed that the HFRS incidence was associated with the density of large animals (cattle, horses, donkeys and mules), sheep density, deer density, and with average temperature, annual precipitation, and average relative humidity. The multivariate analysis revealed that as the deer density increased by 10 head per km^2^, the HFRS incidences increased by 70.7% (P < 0.001). In the combined other nine counties, the results of univariate analysis indicated that the HFRS incidence was significantly associated with the densities of large animals (cattle, horses, donkeys and mules), sheep, and deer, and with average relative humidity values. The multivariate analysis revealed that as the deer density increased by 10 head per km^2^, the HFRS incidences increased by 90.4% (P < 0.001). As the average relative humidity increased by 1%, the HFRS incidences increased by 7.1% (P < 0.001) (Table 2).

**Table 2:** Factors affecting temporal trends in HFRS incidence for Shuangyang County, and for the combined other nine counties, Changchun

**County**	**Variable (unit)**	**Univariate analysis**		**Multivariate analysis**	
		**PC (95% CI)**	**P-value**	**PC (95% CI)**	**P-value**
**Shuangyang**	**Livestock husbandry**				
	Large animals (10 h/km^2^)	45.0 (42.2, 47.8)	< 0.001	-	-
	Sheep density (10 h/km^2^)	1154.0 (978.0, 1358.8)	< 0.001	-	-
	Deer density (10 h/km^2^)	70.7 (65.8, 75.7)	< 0.001	70.7 (65.8, 75.7)	< 0.001
	**Climate factors**				
	Temperature (1°C)	61.9 (49.9, 74.8)	< 0.001	-	-
	Precipitation (1 mm)	-35.2 (-37.8, -32.4)	< 0.001		
	Relative humidity (%)	-3.2 (-3.8, -2.5)	< 0.001	-	-
**Other nine counties**	**Livestock husbandry**				
	Large animals (10 h/km^2)^	26.8 (24.9, 28.8)	< 0.001	-	-
	Sheep density (10 h/km^2^)	68.6 (63.0, 74.5)	< 0.001	-	-
	Deer density (1 h/km^2^)	76.4 (69.0, 84.1)	< 0.001	90.4 (81.6, 99.6)	< 0.001
	**Climate factors**				
	Temperature (1°C)	4.8 (0.02, 9.7)	0. 049	-	-
	Precipitation (1 mm)	-1.1 (-1.6, -0.7)	< 0.001	-	-
	Relative humidity (%)	-6.1 (-7.6, -4.5)	< 0.001	7.1 (5.1, 9.0)	< 0.001

Time-series Poisson regression analysis results.

## Discussion

Since 1998, the HFRS incidence in Changchun has increased dramatically. The HFRS hotspot has been located at Shuangyang County in southeastern Changchun. Two epidemic phases were identified by examining the seasonal pattern of HFRS incidence. One phase was characterized by a single epidemic peak at the end of each year during 1988–1997. The other phase had two separate epidemic peaks, which occurred at the end and the beginning of each year since 1998. The peak at the beginning of each year could be associated with the emergence of SEOV. The transmission of hantaviruses through *A. agrarius* mice peaks during the fall-winter period, while *R. norvegicus* rat-associated infections mainly occur in spring [11,12,16,19]. Given the well-documented evidence, and the two-phase pattern observed in the present study, we infer that the HFRS phase I (1988–1997) pattern was primarily caused by HTNV infections. HFRS phase II (since 1998) pattern was caused by both HTNV and SEOV infections. Noticeably, the newly established and re-emerging HFRS endemic areas in mainland China since the 1990s that included Beijing City, Shandong Province, Huludao City, and Changchun, could be associated with SEOV (e.g. peridomestic rodents-associated) infections indicated by a characteristic spring peak in human cases [10,16,20]. In mainland China, antigen-positive *A. agrarius*, *Apodemus peninsulae*, and *R. norvegicus* rats are predominant in rural, forest, and urban areas, respectively [12]. The results suggest that prioritizing control efforts on peridomestic rodent populations in residential areas in spring and on sylvatic rodent populations in late autumn and early winter might provide an effective means of targeting the specific hantaviruses that cause HFRS.

The Poisson regression analysis revealed that livestock husbandry, especially deer cultivation, was significantly associated with spatial and temporal distributions of HFRS incidence. Hantaviruses are primarily transmitted from rodent hosts to humans by aerosols generated from contaminated excreta (e.g., urine and feces) of rodents, and to a lesser extent by contaminated food or rodent bites [7]. The incidence of HFRS is mainly determined by infection rate and distribution of rodent hosts; host distribution is affected by the natural habitat structure (e.g., human buildings, landscape composition, landscape configuration, annual mean temperature, and seasonal variation) [21,22]. Environmental changes that include climate, human agriculture, and social-economic conditions can lead to a change in virus transmission risk from infected rodents to humans [3,23]. The results of a study in Elunchun and Molidawahaner counties of Inner Mongolia indicated that climate variability (including rainfall, land surface temperature, relative humidity and the multivariate El Niño Southern Oscillation index) had a significant role in HFRS transmission in northeastern China [24]. The results of a study on hantavirus pulmonary syndrome in the Four Corners region of United States revealed that environmental factors (e.g., the dramatic increase in precipitation associated with the 1992 to 1993 El Niño) could indirectly increase the risk for Sin Nombre virus exposure [25]. Furthermore, heterogeneity in the effects of environmental factors on hantavirus diseases between different areas could result from differences in biotopes, climatic conditions, viruses, and the rodent reservoirs [26]. In this study, time-series Poisson regression analysis was used to account for the complex associations between HFRS incidence and influencing factors during a long period of time. Compared with climatic factors, livestock husbandry (mainly deer density) seemed to have a greater effect on HFRS incidence. The analysis of the factors relating to spatial heterogeneity of HFRS incidence also indicated that deer density could play a role in the distribution of HFRS cases, especially for the SEOV-dominated HFRS that occurred from 1998 to 2012. Livestock husbandry has been one of key driving forces of emerging infectious diseases and has also modified the transmission of endemic infections [27,28]. Traditional livestock husbandry was reinstituted at the end of the 1980s and extended into the mid-1990s in Changchun. Deer cultivation and production of deer-related products has been prominent in northeastern China, especially in Changchun. As the numbers of livestock increased, more farms were established and more feed was needed. This additional feed has provided habitats and food for rodents. Farming activities that are related to livestock husbandry can also increase the probability of exposure to infective rodents [12,29]. The authors of a case–control study in Sichuan Province in China suggested that farming activities related to livestock husbandry (e.g., having haystacks for livestock in the yard or indoors in barns) can increase the risk of HFRS transmission [29]. Rodents on pig and chicken farms are considered to be a potential threat to human and animal health, and at least 20 pathogens (e.g., hantaviruses and *Leptospira* species) can be found on the rodents caught on pig or chicken farms and in the surrounding area [30]. Livestock husbandry could have an intermediate role in HFRS incidence by affecting rodent populations and increasing human exposure to aerosols and secretions from infected rodents. The observed association between HFRS incidence and deer cultivation was statistically significant, but it does not prove causality. Further field investigations on rodent population density and hantavirus infection status in rodents living around livestock farms are needed to improve our understanding of the underlying mechanism.

The results of this study indicated that the HFRS incidence was associated with yearly average relative humidity. Rodent population size increase rapidly in response to favorable weather conditions [31]. The relationship between rodent population dynamics and meteorological factors is complex, and varies by rodent species and climate regions [10,24,32]. These complicated relationships may have different effects on disease transmission, and usually display nonlinear patterns between diseases and climate factors in a large geographical area. The association between HFRS and relative humidity that was found in this study was consistent with the result reported for a nearby area of Changchun [24]. Higher humidity levels affect the infectivity and stability of the virus in the ex vivo environment [33,34]. However, the underlying mechanisms for the positive correlations between HFRS incidence and relative humidity are not yet clear. Our study results indicated that livestock cultivation rather than climatic factors likely has a more important role in the emergence of HFRS outbreaks in Changchun. In addition, we have not found significant associations between the spatial distribution of HFRS incidence and land cover. It is likely that the land cover differences between counties in this study area were not significant.

The reemergence of HFRS was also affected by multiple factors including prevention measures, and other human activities [35-37]. As the official documents preserved in Changchun CDC indicate, enhanced measures that include epidemiological surveillance, deratization, vaccination and health education have been implemented for prevention and control of the disease since incidence increased at the end of 1990s. These measures were improved further in 2005. The vaccine mostly used was a univalent HTNV vaccine. It was used in Shuangyang, Jiutai, and Yushu from 1999 to 2004, in the town populations with high HFRS incidence (>5/100,000 population). A bivalent purified inactivated vaccine against HTNV and SEOV infections has been used in these areas since 2005. A significant decline in HFRS incidence has occurred since 2006 (Figure 2). One limitation of this study was that we did not examine vaccination efficacy, but this omission was the result of a lack of detailed information. Another study limitation was that we could not confirm the shifts in virus transmission because the information on the classification of hantaviruses in HFRS patients and infected rodents was missing. Future studies on the classification of hantaviruses are needed. The results of this study indicated that prevention and control of HFRS in Changchun will benefit from targeting control measures at *R. norvegicus* populations*,* especially at livestock husbandry sites. The need for vaccination of farmers involved in livestock husbandry in early spring should also be emphasized.

## Conclusions

HFRS incidence has significantly increased in Changchun since 1998. This increase included a seasonal shift in HFRS incidence from one epidemic peak in the end of the year to dual epidemic peaks in the beginning and in the end of the year. The increase in incidence was significantly associated with livestock husbandry and climate factors, but was especially associated with deer cultivation. Our results indicated that the spatial and temporal variations in HFRS incidence could be associated with local livestock husbandry. These findings will be helpful for the prevention of HFRS, because they indicate that prevention and control measures should be targeted at the sites related with livestock husbandry.

## Competing interests

All authors declare they have no actual or potential competing financial interest. The funders had no role in the study design, data collection and analysis, decision to publish, or preparation of the manuscript.

## Authors’ contributions

WCC, YWL,YQY and LQF designed the study; JW and DDW collected data; JW, DDW, XLL, SJDV and LQF performed the statistical analyses and outcome assessments, and wrote the paper. JZ, YZ, BW and LY also contributed to the statistical analyses and the outcome assessments. All authors read and approved the final manuscript.

## Pre-publication history

The pre-publication history for this paper can be accessed here:

http://www.biomedcentral.com/1471-2334/14/301/prepub

## References

[B1] Bi ZQ Formenty PBH Roth CE Hantavirus infection: a review and global update J Infect Dev Ctries 2008 14 1 3 23 19736383 10.3855/jidc.317

[B2] Kariwa H Kumiko Y Arikawa J Hantavirus infections in East Asia Comparative Med 2007 14 341 356 10.1016/j.cimid.2007.05.011 17655929

[B3] Jonsson CB Figueiredo LTM Vapalahti O A global perspective on Hantavirus ecology, epidemiology, and disease Clin Microbiol Rev 2010 14 2 412 441 10.1128/CMR.00062-09 20375360 PMC2863364

[B4] Vapalahti O Mustonen J Lundkvist A Henttonen H Plyusnin A Vaheri A Hantavirus infections in Europe Lancet Infect Dis 2003 14 653 661 10.1016/S1473-3099(03)00774-6 14522264

[B5] Zhang YZ Xiao DL Wang Y Wang HX Sun L Tao XX Qu YG The epidemic characteristics and preventive measures of hemorrhagic fever with renal syndrome in China Chin J Epidemiol 2004 14 466 469 in Chinese 15231118

[B6] Luo CW Chen HX Study on the factors influenced epidemic of hemorrhagic fever with renal syndrome Chin J Vector Biol Control 2003 14 451 454 in Chinese

[B7] Clement JP Hantavirus Antiviral Res 2003 14 121 127 10.1016/S0166-3542(02)00205-X 12615308

[B8] Heyman P Vaheri A Lundkvist A Avsic-Zupanc T Hantavirus infectious in Europe: from virus carries to a major public-health problem Expert Rev Anti Infect Ther 2009 14 2 205 217 10.1586/14787210.7.2.205 19254169

[B9] Hart CA Bennett M Hantavirus infections: epidemiology and pathogenesis Microbes Infect 1999 14 1229 1237 10.1016/S1286-4579(99)00238-5 10580279

[B10] Fang LQ Wang XJ Liang S Li YL Song SX Zhang WY Qian Q Li YP Wei L Wang ZQ Yang H Cao WC Spatiotemporal trends and climatic factors of hemorrhagic fever with renal syndrome epidemic in Shandong Province, China PLoS Negl Trop Dis 2010 14 8 e789 10.1371/journal.pntd.0000789 20706629 PMC2919379

[B11] Chen HX Qiu FX Epidemiological surveillance on the hemorrhagic fever with renal syndrome in China Chin Med J 1993 14 857 863 7908258

[B12] Chen HX Qiu FX Zhao XQ Luo CW Li XQ Characteristics of the distribution of epidemic season of hemorrhagic fever with renal syndrome in different regions and different years in China Chin J Exp Clin Virol 1994 14 197 203 In Chinese

[B13] Zhang YZ Zou Y Fu ZF Plyusnin A Hantavirus infections in humans and animals, China Emerg Infect Dis 2010 14 1195 1203 10.3201/eid1608.090470 20678311 PMC3298307

[B14] Wu YP Huang B Hu GW Yao LS Xun WM Analysis on epidemiologic feature of HFRS in Jilin province Chin J Ctrl Endem Dis 2004 14 164 165 in Chinese

[B15] Surveillance Protocol of Hemorrhagic Fever With Renal Syndrome in Chinahttp://www.nhfpc.gov.cn/zhuzhan/zcjd/201304/f0ec588ed89e43739e507b9573851060.shtml

[B16] Zhang YZ Zhang FX Wang JB Zhao ZW Li MH Chen HX Zou Y Plyusnin A Hantaviruses in rodents and humans, Inner Mongolia Autonomous Region, China Emerg Infect Dis 2009 14 885 891 10.3201/eid1506.081126 19523286 PMC2727351

[B17] Liu JY Zhang ZX Xu XL Kuang WH Zhou WC Zhang SW Li RD Yan CZ Yu DS Wu SX Nan J Spatial patterns and driving forces of land use change in China during the early 21st century J Geogr Sci 2010 14 483 494 10.1007/s11442-010-0483-4

[B18] Kuang WH Liu JY Zhang ZX Lu DS Xiang B Spatiotemporal dynamics of impervious surface areas across China during the early 21st century Chin Sci Bull 2013 14 14 1691 1701 10.1007/s11434-012-5568-2

[B19] Kim YS Ahn C Han JS Kim S Lee JS Lee PW Hemorrhagic fever with renal syndrome caused by the Seoul virus Nephron 1995 14 419 427 10.1159/000188762 8587622

[B20] Guan P Huang DS He M Shen TF Guo JQ Zhou BS Investigating the effects of climatic variables and reservoir on the incidence of hemorrhagic fever with renal syndrome in Huludao city, China: a 17-year data analysis based on structure equation model BMC Infect Dis 2009 14 109 10.1186/1471-2334-9-109 19583875 PMC2720978

[B21] Wei L Qian Q Wang ZQ Class GE Song SX Zhang WY Li XJ Yang H Wang XJ Fang LQ Cao WC Using geographic information system-based ecologic niche models to forecast the risk of hantavirus infection in Shandong Province, China Am J Trop Med Hyg 2011 14 3 497 503 10.4269/ajtmh.2011.10-0314 21363991 PMC3042829

[B22] Langlois JP Fahrig L Merriam G Artsob H Landscape structure influences continental distribution of hantavirus in deer mice Landscope Ecol 2001 14 255 266 10.1023/A:1011148316537

[B23] Semenza JC Menne B Climate change and infectious diseases in Europe Lancet Infect Dis 2009 14 365 375 10.1016/S1473-3099(09)70104-5 19467476

[B24] Zhang WY Guo WD Fang LQ Li PB Glass GE Jiang JF Sun SH Qian Q Liu W Yan L Yang H Tong SL Cao WC Climate variability and hemorrhagic fever with renal syndrome transmission in Northeastern China Environ Health Persp 2010 14 118 915 920 10.1289/ehp.0901504 PMC2920909 20142167

[B25] Engelthaler DC Mosley DG Cheek JE Levy CE Komatsu KK Ettestad P Davis T Tanda DT Miller L Frampton W Porter R Bryan RT Climatic and environmental patterns associated with hantavirus pulmonary syndrome, four corners region, United States Emerg Infect Dis 1999 14 1 87 94 10.3201/eid0501.990110 10081675 PMC2627709

[B26] Xiao H Lin X Gao L Huang C Tian H Li N Qin J Zhu P Chen B Zhang X Zhao J Ecology and geography of hemorrhagic fever with renal syndrome in Changsha, China BMC Infect Dis 2013 14 305 10.1186/1471-2334-13-305 23819824 PMC3708768

[B27] Coker RJ Hunter BM Rudge JW Liverani M Hanvoravongchai P Emerging infectious diseases in southeast Asia: regional challenges to control Lancet 2011 14 599 609 10.1016/S0140-6736(10)62004-1 21269678 PMC7159088

[B28] Bradley CA Altizer S Urbanization and the ecology of wildlife diseases Trends Ecol Evol 2007 14 2 95 102 10.1016/j.tree.2006.11.001 17113678 PMC7114918

[B29] Sun SP Li NX Yang SJ Case–control study on the risk factors for hemorrhagic fever with renal syndrome in Yanyuan county Modern Prev Med 2009 14 828 829 in Chinese

[B30] Backhans A Fellstrom C Rodents on pig and chicken farms: a potential threat to human and animal health Infect Ecol Epidemiol 2012 14 17093 10.3402/iee.v2i0.17093 PMC3426328 22957130

[B31] Kausrud KL Mysterud A Steen H Vik JO Ostbye E Cazelles B Framstad E Eikeset AM Mysterud I Solhøy T Stenseth NC Linking climate change to lemming cycles Nature 2008 14 93 97 10.1038/nature07442 18987742

[B32] Yan L Fang LQ Huang HG Zhang LQ Feng D Zhao WJ Zhang WY Li XW Cao WC Landscape elements and Hantaan virus-related hemorrhagic fever with renal syndrome, People’s Republic of China Emerg Infect Dis 2007 14 1301 1306 10.3201/eid1309.061481 18252099 PMC2857277

[B33] Hardestam J Simon M Hedlund KO Vaheri A Klingstrom J Lundkvist A Ex vivo stability of the rodent-borne Hantaan virus in comparison to that of arthropod-borne members of the Bunyaviridae family Appl Environ Microbiol 2007 14 8 2547 2551 10.1128/AEM.02869-06 17337567 PMC1855600

[B34] Vickery WL Bider JR The influence of weather on rodent activity J Mamm 1981 14 1 140 145 10.2307/1380484

[B35] Casico A Bosilkovski M Rodriguez-Morales AJ Pappas G The socio-ecology of zoonotic infections Clin Microbiol Infect 2010 14 336 342 10.1111/j.1469-0691.2010.03451.x 21175957

[B36] Pascual M Dobson A Seasonal patterns of infectious diseases PLoS Med 2005 14 1 e5 10.1371/journal.pmed.0020005 15696215 PMC545198

[B37] Snowden FM Emerging and reemerging diseases: a historical perspective Immunological Rev 2008 14 9 26 10.1111/j.1600-065X.2008.00677.x PMC7165909 18837773

